# A p21-Activated Kinase Is Required for Conidial Germination in Penicillium marneffei

**DOI:** 10.1371/journal.ppat.0030162

**Published:** 2007-11-02

**Authors:** Kylie J Boyce, Alex Andrianopoulos

**Affiliations:** Department of Genetics, University of Melbourne, Victoria, Australia; Johns Hopkins, United States of America

## Abstract

Asexual spores (conidia) are the infectious propagules of many pathogenic fungi, and the capacity to sense the host environment and trigger conidial germination is a key pathogenicity determinant. Germination of conidia requires the de novo establishment of a polarised growth axis and consequent germ tube extension. The molecular mechanisms that control polarisation during germination are poorly understood. In the dimorphic human pathogenic fungus Penicillium marneffei, conidia germinate to produce one of two cell types that have very different fates in response to an environmental cue. At 25 °C, conidia germinate to produce the saprophytic cell type, septate, multinucleate hyphae that have the capacity to undergo asexual development. At 37 °C, conidia germinate to produce the pathogenic cell type, arthroconidiating hyphae that liberate uninucleate yeast cells. This study shows that the p21-activated kinase *pakA* is an essential component of the polarity establishment machinery during conidial germination and polarised growth of yeast cells at 37 °C but is not required for germination or polarised growth at 25 °C. Analysis shows that the heterotrimeric G protein α subunit GasC and the CDC42 orthologue CflA lie upstream of PakA for germination at both temperatures, while the Ras orthologue RasA only functions at 25 °C. These findings suggest that although some proteins that regulate the establishment of polarised growth in budding yeast are conserved in filamentous fungi, the circuitry and downstream effectors are differentially regulated to give rise to distinct cell types.

## Author Summary

Many fungal infections are initiated by the entry of dormant fungal spores into their host. Once inside the host these dormant spores must reactivate (germinate) for the infection to proceed. Productive infections necessitate that the fungus grow and divide within the host, which makes understanding the mechanisms that control germination crucial to developing preventative or prophylactic treatments for fungal infections. The molecular mechanisms that control spore germination are poorly understood and studies of the opportunistic fungal pathogen Penicillium marneffei have shown that a group of highly conserved signalling and cell polarity factors, known as small GTPases, play important roles in germination and other aspects of morphogenesis. In this study we have shown that a downstream target of these small GTPases, a p21-activated kinase plays a crucial role in germination at the host temperature of 37 °C but not at 25 °C. This is the first component of germination, which shows temperature-dependent effects and provides insights into the different mechanisms used by fungal pathogens to infect their host or to grow saprophytically in non-host environments.

## Introduction

The generation of an axis of cell polarity is central to the activity of many cells and the establishment of a wide variety of cell morphologies. It relies on the ability to mark different cellular regions by specific protein localisation. The establishment of polarised growth requires selection of a site to which proteins and components of the cytoskeleton are recruited. Growth is then directed specifically to this site via targeted cellular trafficking and concomitant cell wall deposition. Fungi are small eukaryotes that exhibit highly polarised growth patterns and provide excellent models for the study of the molecular mechanisms underlying cell polarity. Saccharomyces cerevisiae establishes a polarised axis of growth during the processes of budding cell division, schmoo formation during mating, and pseudohyphal growth. Under conditions of nitrogen starvation, S. cerevisiae diploid cells undergo pseudohyphal growth, a morphological switch that involves changes in cell shape and division [1]. Pseudohyphal growth requires the initiation of polarised growth for cellular elongation and is under the control of two signaling pathways: a cyclic adenosine monophosphate (cAMP)/protein kinase A (PKA) pathway and a mitogen-activated protein kinase (MAPK) cascade. The cAMP/PKA pathway is activated by a glucose/sucrose sensitive receptor Gpr1p, which activates the G protein Gpa2p (α subunit), which in turn is inhibited by the novel kelch-Gβ subunits Gpb1/2p, a third subunit Gpg1p, and a negative regulator Rgs2p [2–8]. The Gpa2-Gpb1/2 complex regulates the cAMP/PKA pathway directly in association with the Ras2p GTPase and the RasGAP neurofibromin homologues Ira1/2p [6–7, 9]. Ras2p can also activate the MAPK cascade by activating the guanine nucleotide exchange factor Cdc24p, which catalyzes the guanosine diphosphate (GDP) to guanosine triphosphate (GTP) exchange of the Rho GTPase Cdc42p [1,10,11]. GTP-bound Cdc42p is required to initiate actin polarisation and recruits and activates additional proteins required for polarised growth such as septins, myosins, and the p21-activated kinase (PAK) Ste20p (reviewed in [12]). In turn, Ste20p activates the MAPK cascade by phosphorylating Ste11p (MAPKKK); Ste11p phosphorylates Ste7p (MAPKK), which then activates the Kss1p MAPK [13–15].

Filamentous fungi exhibit a highly polarised axis during vegetative hyphal growth, asexual (conidiation) and sexual (mating) development, and initiation of polarised filamentous growth during the germination of asexual (conidia) and sexual (ascospores) spores. Spherical conidia germinate under favorable environmental conditions by initially growing isotropically. This growth is followed by the establishment of a de novo axis of polarised growth to allow a germ tube to emerge [16–18]. Conidial germination is a central aspect of fungal cell propagation, initiating the formation of the extensive radiating hyphal network necessary for colonization of substrates from a dormant conidium. Conidia are also the infectious propagules of many pathogenic fungi, and germination of conidia in the lung or leaves of potential hosts is likely to be a key pathogenicity determinant. Studies in the filamentous fungus Aspergillus nidulans have shown that the cAMP-PKA and Ras pathways play a role [18–20]. However, the molecular mechanisms governing germination of fungal conidia are not well understood in any system.

Penicillium marneffei is an opportunistic human pathogen with a thermally regulated dimorphic switch. At 25 °C, in the saprophytic growth phase, conidia germinate to produce highly polarised, septate, branched, multinucleate hyphae. Conidia can also germinate at 37 °C to produce polarised arthroconidiating hyphae, in which nuclear division and septation are coupled, double septa are laid down, and fragmentation occurs along this plane to liberate uninucleate yeast cells that consequently divide by fission [21]. The yeast cells are the pathogenic growth form and multiple yeast cells are observed in the pulmonary alveolar macrophages of infected individuals. P. marneffei infection is thought to occur through inhalation of conidia, which bind to the laminin in the broncholalvelolar epithelia. Conidia are then ingested by pulmonary alveolar macrophages and germinate, generating the uninucleate yeast cells [21]. Therefore in *P. marneffei,* conidial germination can lead to two very different morphological programs in response to different temperatures.

Some of the core components regulating polarised growth establishment in S. cerevisiae are conserved during polarity establishment in germinating conidia of filamentous fungi. The P. marneffei Gpa2p homologue, encoded by *gasC*, is required during conidial germination to produce hyphae*.* Deletion of *gasC* results in delayed germination, whereas expression of a dominant activating allele shows a significantly accelerated germination rate [22]. Likewise, the P. marneffei Ras homologue, RasA, is required for conidial germination where expression of either a dominant negative or activated allele results in a germination delay and conidia with abnormal isotropic growth [23]. Expression of either a dominant negative or constitutively active allele of the *P. marneffei CDC42* homologue (*cflA*) results in a decrease or increase in the rate of germination at 25 °C, respectively [24]. The role of GasC, RasA, and CflA during conidial germination in P. marneffei suggests that the core components regulating polarised growth establishment in S. cerevisiae may be conserved during polarity establishment in germinating conidia of filamentous fungi. To investigate if any of the downstream effectors of this core pathway are also conserved in function, a PAK *STE20* homologue (designated *pakA*) was identified and deleted in P. marneffei. Characterization of *pakA* in P. marneffei has shown that this gene is essential for conidial germination at 37 °C and for polarised growth of yeast cells and is downstream of both a heterotrimeric G protein and Cdc42 pathway. In contrast, PakA plays only a minor role during germination of conidia at 25 °C and is not required for polarised growth of hyphae. Germination in this case is controlled by a heterotrimeric G protein–Ras-Cdc42 pathway. These data suggest that although some proteins that regulate the establishment of polarised growth in budding yeast and filamentous fungi may be conserved, the downstream effectors are likely to be different or regulated differently to give rise to the distinct cell types in these two modes of growth.

## Results

### Cloning the *STE20* p21-Activated Kinase Orthologue from P. marneffei

An A. nidulans sequence was identified from the genome sequence (http://www.broad.mit.edu/annotation/genome/aspergillus_group/MultiHome.html) with strong homology to Candida albicans Cst20p (67% identity, 81% similarity) and other PAKs, including S. cerevisiae Ste20p (71% identity, 81% similarity, accession number AAA35039). Primers were designed to amplify the sequence encoding the conserved kinase domain and a PCR product was generated using A. nidulans genomic DNA. The *A. nidulans STE20* homologous sequence was used to screen a P. marneffei genomic library at low stringency. Five positive clones were identified, which fell into two classes based on restriction enzyme digestion patterns. A 6.4 kb *NotI/BglII* hybridizing fragment from one of these classes was subcloned into *NotI/BamHI* digested pBluescript II SK^+^ (pKB5751). Sequencing revealed strong sequence homology to *STE20*-like PAKs from Magnaporthe grisea (78% identity, 89% similarity, accession number AAP93639), Ustilago maydis (74% identity, 83% similarity, accession number AAM97788), and S. cerevisiae (72% identity, 83% similarity). The gene within this clone was subsequently named *pakA*. The *pakA* open reading frame spans 2407 bp and contains seven exons and six introns. The predicted protein is 642 amino acids in length and contains a Cdc42/Rac interactive binding (CRIB) domain at positions 98–115, a predicted kinase domain at 361–612, and a Gβ binding domain at 619–629. Preliminary analysis of the second class of clones revealed that the gene within these clones has strong sequence homology to *CLA4*-like PAKs.

RNA was isolated from vegetative hyphae grown for 2 d in liquid medium at 25 °C, asexual development (conidiation) cultures grown for 4 d on solid medium at 25 °C, and yeast cells grown for 8 d in liquid medium at 37 °C. A *pakA* transcript was detected under all conditions. The amount of *pakA* transcript was approximately equivalent under all conditions when compared with the histone H3 control (unpublished data).

### Deletion of *pakA* in P. marneffei

A *pakA* construct, which deleted from −425 to +2030 of *pakA,* was linearised and transformed into P. marneffei strain SPM4 (*niaD1 pyrG1*) and *pyrG^+^* transformants selected. PyrG^+^ transformants were screened by genomic Southern blotting and one strain was isolated that possessed a restriction pattern consistent with replacement of *pakA* by *pyrG* at the genomic locus. The deletion strain was plated on medium containing 5-fluoroorotic acid (5-FOA) to generate a Δ*pakA pyrG* strain (Materials and Methods). This strain was cotransformed with plasmids containing *pakA^+^* and *pyrG^+^* genes and co-transformants confirmed by Southern blot analysis. The transformants contained from 1–7 copies of *pakA*.

In S. cerevisiae, the H345G mutation in the conserved CRIB domain of Ste20p results in a mutant protein that shows reduced interaction with the upstream activator Cdc42p (GTPase) in a two-hybrid assay and loss of correct localisation to sites of polarised growth [25]. The phenotype of the *STE20^H345G^* strain is almost equivalent to the null, indicating that the interaction of Cdc42p and Ste20p is essential for Ste20p function [25]. The equivalent mutation was generated in *P. marneffei pakA* (*pakA^H108G^*) by inverse PCR (Materials and Methods). The *pakA^H108G^* construct was co-transformed with *pyrG^+^* into the Δ*pakA pyrG^−^* strain and transformants selected for PyrG^+^. Co-transformation was confirmed by Southern blot analysis of genomic DNA and four representative transformants with copy numbers ranging from 3 to 12 were selected for further analysis.

### PakA Is Localised to Sites of Polarised Growth

**Figure 1:**
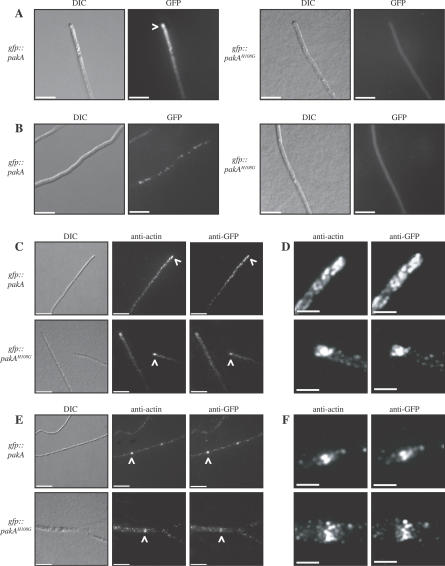
PakA Co-Localises with Actin at Sites of Polarised Growth Localisation of PakA in live cells (A, B) and in immunofluorescently labeled fixed cells (C–F) at 25 °C. (A) The GFP::PakA fusion protein is localised to the hyphal apex at 25 °C (white arrowhead). In contrast, the GFP::PakA^H108G^ fusion protein is visible throughout the cytoplasm. (B) In subapical hyphal cells, the GFP::PakA fusion protein is observed as spots along subapical hyphal cells. This localisation is not observed in the *gfp::pakA^H108G^* strains. (C) The PakA and PakA^H108G^ GFP fusion proteins co-localised with actin at the hyphal tip (indicated by white arrowheads). (D) Enlargement of the stained region indicated by arrowheads in C. (E) At 25 °C the PakA and PakA^H108G^ GFP fusion proteins also co-localise with actin at nascent septation sites (indicated by arrowheads). (F) Enlargement of the stained region indicated by arrowheads in E. Images were captured using DIC or under epifluorescence to detect GFP, anti-actin, or anti-GFP. Scale bars, 20 μm.

The *gfp::pakA* and *gfp::pakA^H108G^* fusion constructs were generated and co-transformed with the *pyrG^+^* gene into the Δ*pakA pyrG* strain to investigate the localisation of PakA and to assess whether the *pakA^H108G^* mutation affects PakA localisation. Transformants were selected for PyrG^+^ and confirmed by Southern blot analysis of genomic DNA. Four transformants of each genotype were selected for further analysis and had copy numbers ranging from 4 to 9 for *gfp::pakA* and from 2 to 20 for *gfp::pakA^H108G^*. The *gfp::pakA* and *gfp::pakA^H108G^* strains were grown on agar-coated slides for 2 and 4 d at 25 °C and 37 °C, respectively. At 25 °C, the GFP::PakA fusion protein was concentrated at the hyphal apex (Figure 1A) and localised as spots along the subapical cells of the hyphae (Figure 1B). In contrast, the GFP::PakA^H108G^ fusion protein was visible as diffuse fluorescence in the cytoplasm but not concentrated at the hyphal apex or as spots along the hyphae (Figure 1A and 1B). At 37 °C, the GFP::PakA fusion protein was concentrated at the apex of arthroconidiating hyphae, although this fluorescence was less intense than that visualized in the same transformants at 25 °C. The GFP::PakA^H108G^ fusion protein was not observed at the apex of arthroconidiating hyphae (unpublished data).

To investigate whether the GFP::PakA fusion protein co-localises with actin at the hyphal apex and at nascent septation sites, immunostaining using mouse anti-actin and rabbit anti-GFP antibodies was performed on two of the *gfp::pakA* and two of the *gfp::pakA^H108G^* strains at both 25 °C and 37 °C. At 25 °C, actin is localised as cortical actin spots along the hyphae and is concentrated at nascent septation sites and the hyphal apex (Figure 1C–1F). The GFP::PakA fusion protein co-localised with actin at all of these locations (Figure 1C–1F). The GFP::PakA^H108G^ fusion protein also co-localised with actin at cortical actin patches, nascent septation sites, and the hyphal apex, in addition to showing diffuse staining throughout the cytoplasm (Figure 1C–1F).

At 37 °C, actin is also localised as cortical patches along the arthroconidiating hyphae, concentrated at nascent septation sites and the apex of arthroconidiating hyphae (Figure S1). The GFP::PakA and GFP::PakA^H108G^ fusion proteins co-localised with actin at all of these sites (Figure S1).

### The Role of *pakA* during Asexual Development and Hyphal Growth at 25 °C

**Figure 2:**
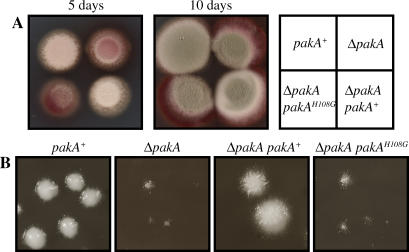
Vegetative Growth Is Reduced in Mutant *pakA* Strains (A) Hyphal growth of the wild-type (*pakA^+^*), Δ*pakA*, Δ*pakA pakA^H108G^*, and Δ*pakA pakA^+^* strains after 5 and 10 d incubation at 25 °C. The Δ*pakA* and Δ*pakA pakA^H108G^* strains show reduced growth as evidenced by the red colony appearance due to significantly reduced aerial hyphal growth after 5 d. After 10 d, all strains are conidiating. (B) Yeast colonies of the wild-type (*pakA^+^*), Δ*pakA*, Δ*pakA pakA^H108G^*, and Δ*pakA pakA^+^* strains after 5 d at 37 °C. The Δ*pakA* and Δ*pakA pakA^H108G^* strains show reduced growth at 37 °C.

At 25 °C, P. marneffei colonies are comprised of highly polarised vegetative hyphae growing on the agar surface and bearing asexual structures (conidiophores) that appear green due to the presence of pigmented asexual spores (conidia) on the conidiophores. After 5 d growth at 25 °C, surface hyphae are visible in the wild-type strain (Figure 2A). The Δ*pakA pakA^+^* strains appeared wild-type after 5 d at 25° C, whereas both the Δ*pakA* and Δ*pakA pakA^H108G^* strains showed a reduction in growth (Figure 2A). Despite the initial reduction in growth, after 10 d all strains were producing conidia. P. marneffei colonies are yeast-like at 37 °C and Δ*pakA pakA^+^* strains were indistinguishable from the wild type when grown for 4 d at 37 °C (Figure 2B). In contrast, the Δ*pakA* and Δ*pakA pakA^H108G^* strains displayed reduced growth at 37 °C (Figure 2B).

**Figure 3:**
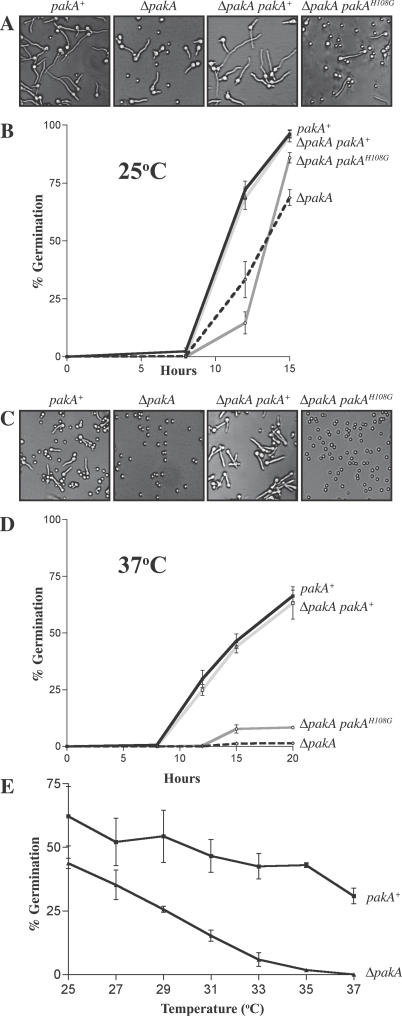
Mutation of *pakA* Affects Conidial Germination at 25 °C and 37 °C (A) Conidia from the wild-type (*pakA^+^*), Δ*pakA*, Δ*pakA pakA^+^*, and Δ*pakA pakA^H108G^* strains were grown in liquid medium for 12 h at 25 °C (A) or 37 °C (C). The percentage of germinated conidia was measured after 8, 12, or 15 h incubation at 25 °C (B) or after 8, 12, 15, and 20 h at 37 °C (D). One representative strain of each genotype is shown. Three independent experiments were performed and standard error of the mean bars are shown. (A) Compared with wild-type and the complemented Δ*pakA pakA^+^* strain, the Δ*pakA* and Δ*pakA pakA^H108G^* strains have a larger number of ungerminated conidia visible after 12 h incubation at 25 °C. (B) In contrast to the wild-type (*pakA^+^*) and Δ*pakA pakA^+^* strain, the Δ*pakA* and Δ*pakA pakA^H108G^* strains show slightly delayed germination at 25 °C. (C) Unlike wild-type (*pakA^+^*) and the complemented Δ*pakA pakA^+^* strain, conidia of the Δ*pakA* and Δ*pakA pakA^H108G^* strains remain predominately ungerminated after 12 h incubation at 37 °C. (D) The Δ*pakA* and Δ*pakA pakA^H108G^* strains show a severe germination defect at 37 °C, with only a small proportion of conidia germinating. (E) Conidia of the wild-type and Δ*pakA* strain were incubated in liquid medium at 2 °C increments from 25 °C to 37 °C. The percentage of germination was assessed after 20 h. The Δ*pakA* strain shows a gradual decrease in the percentage of germinated conidia as the temperature increases.

Both the Δ*pakA* and Δ*pakA pakA^H108G^* strains exhibit a delay in growth after 5 d at 25 °C (Figure 2A). One explanation for this difference could be a delay in the germination of conidia. The kinetics of germination were measured at 25 °C in the wild-type (*pakA^+^*), Δ*pakA*, Δ*pakA pakA^+^*, and two Δ*pakA pakA^H108G^* strains by counting the number of ungerminated versus germinated conidia (conidia with a visible germ tube) in a population of 100 in three independent experiments after 8, 12, or 15 h incubation in liquid media (Table S1). The complemented Δ*pakA* strain (Δ*pakA pakA^+^*) is indistinguishable from wild type (Figure 3A and 3B). The Δ*pakA* and Δ*pakA pakA^H108G^* strains show a minor delay in germination at 25 °C (Figure 3A and 3B).

To investigate if the deletion of *pakA* or the *pakA^H108G^* mutation results in aberrant hyphal morphology or asexual development at 25 °C the wild-type, Δ*pakA*, Δ*pakA pakA^+^*, and Δ*pakA pakA^H108G^* strains were grown on agar-coated slides for 2 or 4 d at 25 °C, stained with calcofluor (to observe cell walls) or 4′6-diamidino-2-phenylindole (DAPI; to observe nuclei), and examined microscopically. After 2 d at 25 °C, wild-type P. marneffei grows as septate, branched hyphae of which subapical cells are predominately uninucleate and apical cells are multinucleate. The Δ*pakA*, Δ*pakA pakA^+^*, and Δ*pakA pakA^H108G^* strains were indistinguishable from wild type after 2 d, with normal morphology, septation, branching, and nuclear index. After 4 d at 25 °C, wild-type P. marneffei begins to undergo asexual development, with the production of a specialized stalk from which differentiated cells are produced sequentially in a budding fashion: metulae bud from the stalk, phialides bud from metulae, and uninucleate conidia bud from phialides. The Δ*pakA*, Δ*pakA pakA^+^*, and Δ*pakA pakA^H108G^* strains produced conidiophores with wild-type morphology (unpublished data).

### *pakA* Is Essential for Conidial Germination and Correct Yeast Cell Morphogenesis at 37 °C

In contrast to the wild-type and Δ*pakA pakA^+^* strains, the Δ*pakA* and Δ*pakA pakA^H108G^* strains displayed reduced growth rates at 37 °C (Figure 2B). To assess if the basis of this difference is because *pakA* is required during the germination of conidia or during yeast morphogenesis and growth at 37 °C, the wild-type (*pakA^+^*), Δ*pakA*, Δ*pakA pakA^+^*, and Δ*pakA pakA^H108G^* strains were inoculated on agar-coated slides and incubated for 4 d at 37 °C. It was immediately apparent that the Δ*pakA* and Δ*pakA pakA^H108G^* strains possessed a severe germination defect and almost all of the conidia remained ungerminated. The germination kinetics were measured by counting the number of ungerminated versus germinated conidia (conidia with a visible germ tube) in a population of 100 in three independent experiments after 8, 12, 15, or 20 h in liquid medium (Table S2). Germination is slower and germlings appear fatter at 37 °C compared with 25 °C (Figure 3A and 3C). In contrast to wild-type and the Δ*pakA pakA^+^* strains, both the Δ*pakA* strain and the Δ*pakA pakA^H108G^* strains showed a severe defect in germination (Figure 3C and 3D). Despite the majority of conidia remaining ungerminated in the Δ*pakA* and Δ*pakA pakA^H108G^* strains, a small proportion do germinate, and it is presumably these cells that go on to establish the colony.

**Figure 4:**
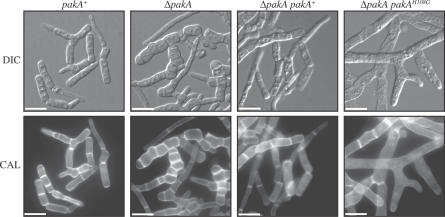
*pakA* Strains Show Aberrant Yeast Morphogenesis at 37 °C The wild-type (*pakA^+^*), Δ*pakA*, Δ*pakA pakA^+^*, and Δ*pakA pakA^H108G^* strains were grown in liquid BHI medium at 37 °C for 6 d. Wild type (*pakA^+^*) produces arthroconidiating hyphae and a mixture of fragmented and separated yeast cells. The Δ*pakA pakA^+^* strains are indistinguishable from wild type. The Δ*pakA* mutant produces swollen arthroconidiating hyphae with increased septation. Fewer yeast cells, which are also swollen and highly septate, are produced in the Δ*pakA* mutant. The Δ*pakA pakA^H108G^* strains produce swollen arthroconidiating hyphae that have reduced septation, and there are reduced numbers of yeast cells. Images were captured using DIC or with epifluorescence to observe calcofluor stained cell walls (CAL). Scale bars, 20 μm.

Growth of the *pakA* strains in liquid medium showed that the small proportion of conidia that germinate go on to form arthroconidiating hyphae that fragment at septation sites to liberate uninucleate yeast cells, just like the wild type. The wild-type (*pakA^+^*), Δ*pakA*, Δ*pakA pakA^+^*, and Δ*pakA pakA^H108G^* strains were grown in liquid brain heart infusion (BHI) for 6 d at 37 °C and cells were stained with calcofluor or DAPI and observed microscopically (Figure 4). In wild type, the culture consists of a mixture of fragmented arthroconidiating hyphae and uninucleate yeast cells. The Δ*pakA* strain produced swollen arthroconidiating hyphae and yeast cells with increased septation (Figure 4) but normal nuclear index. These defects were complemented when the strain was transformed with *pakA^+^*. The Δ*pakA pakA^H108G^* strains also produced swollen arthroconidiating hyphae and yeast cells, but the phenotype was more severe than the Δ*pakA* strain, with very few yeast cells produced and—in contrast to the Δ*pakA* strain and wild type—a decrease in the septation index (Figure 4). Therefore, the *pakA^H108G^* allele has an inhibitory activity on septation.

### PakA Is Required for Conidial Germination In Vivo

**Figure 5:**
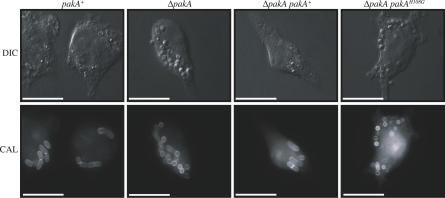
PakA Is Required for Conidial Germination In Vivo LPS activated J774 murine macrophages infected with conidial suspensions of the wild-type (*pakA^+^*), Δ*pakA*, Δ*pakA pakA^H108G^*, and Δ*pakA pakA^+^* strains. After 24 h, numerous yeast cells dividing by fission were observed in macrophages infected with wild-type (*pakA^+^*) or Δ*pakA pakA^+^* conidia. In contrast, conidia of both the Δ*pakA* and Δ*pakA pakA^H108G^* strains remain predominately ungerminated in infected macrophages. Images were captured using DIC or with epifluorescence to observe calcofluor stained fungal cell walls (CAL). Scale bars, 20 μm.

P. marneffei infection occurs through the inhalation of conidia. The conidia are ingested by host pulmonary alveolar macrophages where they germinate into unicellular yeast cells that divide by fission. Multiple yeast cells are seen in the pulmonary alveolar macrophages and peripheral blood mononuclear cells of infected individuals [21]. To investigate if perturbing conidial germination will influence pathogenicity, the ability of the mutant *pakA* strains to germinate into pathogenic yeast cells was investigated. LPS activated J774 murine macrophages were infected with no conidia or with conidia of the wild-type (*pakA^+^*), Δ*pakA*, Δ*pakA pakA^H108G^*, or Δ*pakA pakA^+^* strains (Materials and Methods). After 24 h, numerous yeast cells dividing by fission were observed in macrophages infected with wild-type (*pakA^+^*) or Δ*pakA pakA^+^* conidia (Figure 5). Only 16.3 ± 2.50% or 21.5 ± 2.07% of wild-type (*pakA^+^*) or Δ*pakA pakA^+^* conidia, respectively, remained ungerminated in infected macrophages. In contrast, conidia of the Δ*pakA* or Δ*pakA pakA^H108G^* strains remained predominately ungerminated in infected macrophages (Figure 5). 60.0 ± 3.80% of Δ*pakA* conidia and 66.1 ± 7.16% of Δ*pakA pakA^H108G^* conidia remained ungerminated in macrophages 24 h post infection. This suggests that PakA is required for conidial germination during infection of host macrophages.

### Differential Regulation of Conidial Germination at 25 °C and 37 °C

The temperature-dependent regulation of conidial germination could be the result of the presence of a temperature-specific factor on which PakA depends or the result of a change in the thermostability of a complex in which PakA operates. To distinguish between these two possibilities, wild-type and Δ*pakA* conidia were incubated in liquid medium at different temperatures ranging from 25 °C to 37 °C, in 2 °C increments, and germination rates were measured (Materials and Methods). In contrast to the wild type, the Δ*pakA* strain showed a gradual decrease in the percentage of germination as the temperature increased, indicating that there is no critical temperature during the switch (Figure 3E). This result supports the latter hypothesis and suggests PakA-dependent thermosensitivity. To investigate if Δ*pakA* conidia at 37 °C are waiting for the signal to germinate or have aborted, Δ*pakA* conidia were incubated at 37 °C for 20 h, then at 25 °C for 20 h. The majority of conidia germinated upon switching to 25 °C, indicating that after 20 h incubation at 37 °C, the Δ*pakA* conidia remained viable (unpublished data).

**Figure 6:**
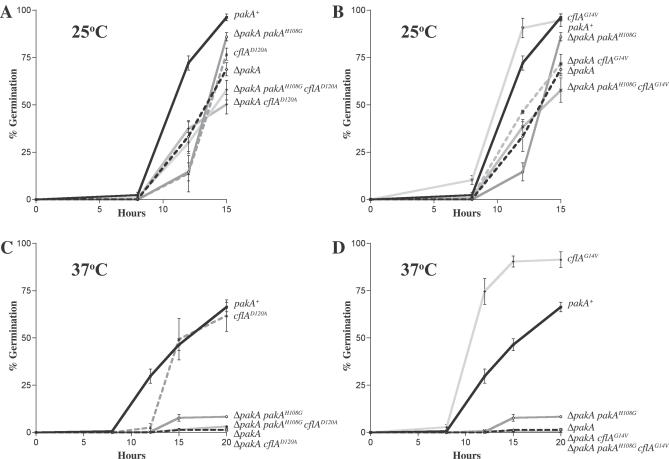
PakA Is Required for CflA-Dependent Germination at Both 25 °C and 37 °C The percentage of germinated conidia was measured in wild-type (*pakA^+^*), *cflA^D120A^*, Δ*pakA*, Δ*pakA pakA^H108G^*, Δ*pakA cflA^D120A^*, and Δ*pakA pakA^H108G^ cflA^D120A^* strains after incubation at 25 °C for 8, 12, and 15 h (A) and at 37 °C for 8, 12, 15, and 20 h (C). The percentage of germinated conidia was measured in wild-type (*pakA^+^*), *cflA^G14V^*, Δ*pakA,* Δ*pakA pakA^H108G^*, Δ*pakA cflA^G14V^*, and Δ*pakA pakA^H108G^ cflA^G14V^* strains after incubation at 25 °C for 8, 12, and 15 h (B) and at 37 °C for 8, 12, 15, and 20 h (D). One representative strain of each genotype is shown. Three independent experiments were performed and standard error of the mean bars are shown. (A, B) At 25 °C, the Δ*pakA* and Δ*pakA pakA^H108G^* strains show a delay in germination when compared with the wild-type control. (A) Compared with the wild type (*pakA^+^*), the *cflA^D120A^* strain shows delayed germination at 25 °C. The Δ*pakA cflA^D120A^* and Δ*pakA pakA^H108G^ cflA^D120A^* strains show delayed germination similar to the Δ*pakA* and Δ*pakA pakA^H108G^* strains. (B) Compared with wild type (*pakA^+^*), the *cflA^G14V^* strain shows accelerated germination. The Δ*pakA cflA^G14V^* and Δ*pakA pakA^H108G^ cflA^G14V^* strains show delayed germination similar to the Δ*pakA* and Δ*pakA pakA^H108G^* strains. (C, D) At 37 °C, the Δ*pakA* and Δ*pakA pakA^H108G^* strains display a dramatic reduction in the number of germinated conidia when compared with wild type (*pakA^+^*). (C) Compared with the wild type (*pakA^+^*), the *cflA^D120A^* strain shows delayed germination at 37 °C. The Δ*pakA cflA^D120A^* and Δ*pakA pakA^H108G^ cflA^D120A^* strains show delayed germination similar to the Δ*pakA* strain. (D) In contrast to wild type (*pakA^+^*), the *cflA^G14V^* strain exhibits accelerated germination at 37 °C. The Δ*pakA cflA^G14V^* and Δ*pakA pakA^H108G^ cflA^G14V^* strains show dramatically reduced germination at 37 °C similar to the Δ*pakA* and *pakA^H108G^* strains.

**Figure 7:**
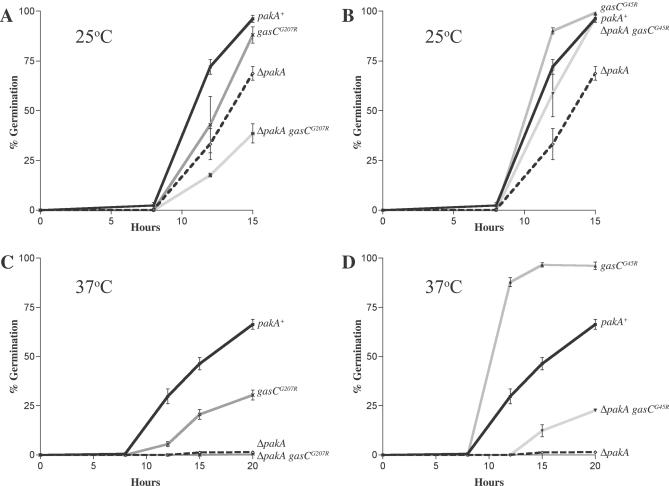
Expression of the Dominant Activated *gasC^G45R^* Allele Partially Suppresses the Germination Defect of the Δ*pakA* Mutant The percentage of germinated conidia was measured in the wild-type (*pakA^+^*), Δ*pakA*, *gasC^G207R^, gasC^G45R^,* Δ*pakA gasC^G207R^,* Δ*pakA gasC^G45R^* strains after incubation at 25 °C for 8, 12, and 15 h (A and B) and at 37 °C for 8, 12, 15, and 20 h (C, D). One representative strain of each genotype is shown. Three independent experiments were performed. Standard error of the mean bars are shown. Compared with wild type (*pakA^+^*), the Δ*pakA* mutant has delayed germination at 25 °C (A, B). (A) At 25 °C, the *gasC^G207R^* strains show delayed germination compared with wild type. Δ*pakA gasC^G207R^* strains display delayed germination that is lower than the Δ*pakA* and *gasC^G207R^* single mutant strains. (B) At 25 °C, the *gasC^G45R^* strains show accelerated germination compared with wild type. The Δ*pakA gasC^G45R^* strains exhibit germination kinetics similar to wild type. (C, D) The Δ*pakA* mutant has a severe germination defect at 37 °C. (C) The *gasC^G207R^* strains show delayed germination at 37 °C. Δ*pakA gasC^G207R^* strains display severely reduced germination similar to the Δ*pakA* strain. (D) Compared with wild type (*pakA^+^*), *gasC^G45R^* strains show accelerated germination at 37 °C. Δ*pakA gasC^G45R^* strains show germination that is intermediate between wild type and the Δ*pakA* strain.

To identify other factors involved in the *pakA*-dependent differences in germination at 25 °C and 37 °C, conidial germination was analyzed in detail at both temperatures in strains carrying mutations in *cflA* (*CDC42* orthologue), *gasC* (*GPA2* orthologue), and *rasA* (*RAS2* orthologue), which have been shown previously to affect germination at 25 °C but which had not been characterized at 37 °C [22–24]. The role of CflA, GasC, and RasA in germination at 37 °C was characterized by assessing the percentage of germinated conidia after 8, 12, 15, and 20 h at both 25 °C and 37 °C in two strains of each genotype (Tables S1 and S2). Two-level nested ANOVA was performed on the data for each time point at both 25 °C and 37 °C to test if germination rates differed significantly between genotypes and also between transformants of the same genotype (Materials and Methods). ANOVA showed there was a significant difference between genotypes in all time points except 0 h. In a few instances, there was variation within transformants of the same genotype (Tables S1 and S2). Strains expressing the dominant negative *cflA^D120A^* allele showed delayed germination at both 25 °C and 37 °C (Figure 6A and 6C). Dominant activated *cflA^G14V^* strains displayed accelerated germination at both 25 °C and 37 °C (Figure 6B and 6D). These results indicate that active CflA promotes conidial germination at both 25 °C and 37 °C. Likewise, the dominant interfering *gasC^G207R^* strains showed delayed germination at both 25 °C and 37 °C (Figure 7A and 7C). The dominant activated *gasC^G45R^* strains showed accelerated germination at 25 °C and 37 °C, suggesting that, like CflA, GasC is required for conidial germination at both 25 °C and 37 °C (Figure 7B and 7D). Both the dominant activated (*rasA^G19V^*) and dominant negative (*rasA^D125A^*) *rasA* strains showed a decrease in germination at 25 °C (Table S1). In contrast, both the dominant negative and dominant activated strains showed wild type germination patterns at 37 °C, suggesting that RasA is required for conidial germination at 25 °C but not at 37 °C (Table S2).

### Genetic Interactions Between *cflA*, *pakA*, and *rasA* during Conidial Germination

To investigate any genetic interaction between *pakA* and *cflA,* double mutants were generated (Δ*pakA cflA^+^,* Δ*pakA cflA^D120A^,* Δ*pakA cflA^G14V^,* Δ*pakA pakA^H108G^ cflA^+^,* Δ*pakA pakA^H108G^ cflA^D120A^*, and Δ*pakA pakA^H108G^ cflA^G14V^*) and germination was characterized by assessing the percentage of germinated conidia after 8, 12, 15, and 20 h at both 25 °C and 37 °C (Tables S1 and S2). It should be noted that multiple copy integrants may result in significant overexpression. Two strains of each genotype were assessed and a single representative strain is shown in Figure 6. ANOVA was performed on the data for each time point at both 25 °C and 37 °C to test if germination rates differed significantly between genotypes and also between transformants of the same genotype (Materials and Methods). ANOVA showed there was a significant difference between genotypes at all time points except 0 h. In a few instances, there was variation within transformants of the same genotype (Tables S1 and S2). The control Δ*pakA cflA^+^* and Δ*pakA pakA^H108G^ cflA^+^* strains showed germination patterns at 25 °C and 37 °C that were indistinguishable from the parental Δ*pakA* and Δ*pakA pakA^H108G^* strains. At 25 °C, the Δ*pakA cflA^D120A^* and Δ*pakA pakA^H108G^ cflA^D120A^* strains displayed delayed conidial germination at rates similar to the single *cflA^D120A^* mutant strains (Figure 6A). At 37 °C, the Δ*pakA cflA^D120A^* and Δ*pakA pakA^H108G^ cflA^D120A^* strains displayed dramatically reduced germination like the single Δ*pakA* and Δ*pakA pakA^H108G^* strains (Figure 6C).

In contrast to the accelerated germination observed in *cflA^G14V^* mutants at 25 °C, which is much faster than wild-type (Figure 6B), the Δ*pakA cflA^G14V^* and Δ*pakA pakA^H108G^ cflA^G14V^* double mutants display slower than wild-type germination at 25 °C, similar to the Δ*pakA* and Δ*pakA pakA^H108G^* single mutant strains (Figure 6B). This suggests that the accelerated germination observed in *cflA^G14V^* strains at 25 °C requires active PakA and an interaction of CflA and PakA via the PakA CRIB domain.

Likewise at 37 °C, in contrast to the accelerated germination observed in *cflA^G14V^* mutants, the Δ*pakA cflA^G14V^* and Δ*pakA pakA^H108G^ cflA^G14V^* double mutants display dramatically reduced germination at rates equivalent to the Δ*pakA* and Δ*pakA pakA^H108G^* single mutant strains (Figure 6D). The inability of the *cflA^G14V^* dominant activated allele to suppress the reduced germination phenotype of the Δ*pakA* and Δ*pakA pakA^H108G^* mutants suggests that at 37 °C, CflA acts upstream of PakA during germination and that an interaction between CflA and the CRIB domain of PakA is required for germination to proceed.

P. marneffei RasA operates upstream of CflA at both 25 °C and 37 °C [23]. The genetic interaction of *pakA* and *rasA* was investigated by generating Δ*pakA rasA^+^*, Δ*pakA rasA^D125A^*, and Δ*pakA rasA^G19V^* double mutants. At 25 °C, the Δ*pakA rasA^+^* strains showed wild-type germination patterns, whereas the Δ*pakA rasA^D125A^* and Δ*pakA rasA^G19V^* mutants showed delayed germination similar to the single *rasA^D125A^* and *rasA^G19V^* mutants (Table S1). At 37 °C, the Δ*pakA rasA^+^*, Δ*pakA rasA^D125A^*, and Δ*pakA rasA^G19V^* double mutants showed severely reduced germination similar to the Δ*pakA* mutant (Table S2).

### GasC Activates Two Pathways Regulating Conidial Germination at 25 °C and 37 °C

To investigate the genetic interaction between *pakA* and *gasC,* double mutants were generated (Δ*pakA gasC^+^,* Δ*pakA gasC^G207R^*, and Δ*pakA gasC^G45R^*). Germination was characterized in five strains of each double mutant genotype by assessing the percentage of germinated conidia after 8, 12, 15, and 20 h at both 25 °C and 37 °C (Tables S1 and S2). ANOVA was performed on the data for each time point at both 25 °C and 37 °C (Materials and Methods) and revealed there was a significant difference between genotypes at all time points except 0 h. In a few instances, there was variation within transformants of the same genotype (Tables S1 and S2). Δ*pakA gasC^+^* strains were indistinguishable from Δ*pakA*. At 25 °C, Δ*pakA gasC^G207R^* strains have delayed germination, which is slower than both the *gasC^G207R^* single mutant strains and the Δ*pakA* mutant (Figure 7A)*.* At 37 °C Δ*pakA gasC^G207R^* strains have a severe reduction in germination similar to Δ*pakA* (Figure 7C). In contrast to the accelerated germination of the *gasC^G45R^* single mutant strains and the delayed germination of the Δ*pakA* mutant, the Δ*pakA gasC^G45R^* double mutant strains show wild-type germination at 25 °C (Figure 7B). In addition, Δ*pakA gasC^G45R^* double mutant strains show germination rates at 37 °C, which are lower than those of wild-type and the *gasC^G45R^* mutants but higher than that of the Δ*pakA* mutant (Figure 7D). This indicates that expression of the *gasC^G45R^* mutant allele partially suppresses the germination defects of Δ*pakA* and suggests that GasC regulates two pathways during germination, one of which is independent of PakA.

## Discussion

### Conserved Proteins Initiate Filamentous Growth but the Downstream Effectors Are Differentially Regulated

**Figure 8:**
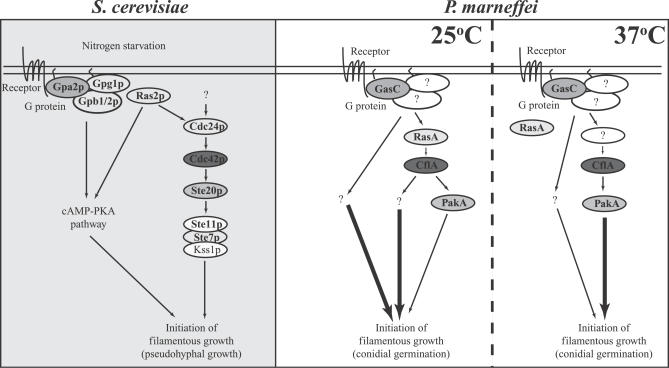
Models for the Regulation of the Initiation of Filamentous Growth in S. cerevisiae and P. marneffei (A) In S. cerevisiae, nitrogen starvation triggers a switch from the unicellular budding growth form to a filamentous growth form (pseudohyphal growth). The activated GTPase Ras2p, activates the GEF Cdc24p, which catalyses the activation of the Rho GTPase Cdc42p. Cdc42p activates the PAK kinase Ste20p, which leads to the activation of a MAPK kinase cascade, which culminates in the activation of the transcription factor Ste12p. The activation of a heterotrimeric G protein is also involved in the initiation of filamentous growth in *S. cerevisiae,* via activation of cAMP signaling. (B) In P. marneffei, GasC (Gpa2p homologue) activates two pathways regulating conidial germination at 25 °C. In one pathway, GasC activates RasA (Ras2p homologue), which activates CflA (Cdc42p homologue). CflA activates PakA (Ste20p homologue) via the CRIB domain and this interaction is required for PakA function. CflA also activates an additional unknown effector at 25 °C, which plays a more important role than PakA in germination. It is possible that GasC directly acts on PakA and does not act upstream of RasA and CflA. B. At 37 °C, GasC also activates two pathways regulating conidial germination. In one pathway, GasC activates CflA, which activates PakA. Similar to 25 °C, at 37 °C CflA activates PakA via the CRIB domain and this interaction is required for PakA function. Unlike 25 °C, at 37 °C RasA is not required for germination, PakA plays a crucial role during the establishment of polarised arthroconidiating hyphal growth, and no additional effector is required.

The establishment of an axis of polarised growth is orchestrated by the asymmetric distribution of cellular components through the localisation and activation of proteins required for growth. Some of the core components regulating polarised growth establishment in S. cerevisiae are conserved both in the genome and functionally during polarity establishment in more complex organisms. However, the question remains as to how multi-cellular organisms generate the greater diversity of distinct cell types with the same set of core components. Unlike small eukaryotes like fungi, larger eukaryotes such as flies and mammals often have an increased number of factors involved in polarity establishment with significant redundancy [26–28]. One possible mechanism is to alter the activity of the key establishment proteins in different cell types, while another is to differentially regulate the effector proteins. In the dimorphic pathogen P. marneffei, the germination of conidia gives rise to two different developmental pathways and cell types. The regulation of conidial germination by CflA (*CDC42* orthologue) and GasC (*GPA2* orthologue) at both 25 °C and 37 °C suggests that the core components regulating polarised growth establishment in S. cerevisiae may be conserved during polarity establishment in germinating conidia of filamentous fungi. However, mutations in *pakA*, a potential downstream effector of *cflA*, result in a dramatic reduction in the rates of germination at 37 °C but not 25 °C. This suggests that in P. marneffei conserved polarity establishment proteins regulate germination, but the downstream effectors are differentially regulated to give rise to distinct cell types. The results suggest a model in which, at 25 °C, GasC activates two pathways regulating conidial germination (Figure 8). In one pathway, RasA activates CflA, which activates PakA—by association via the CRIB domain—and a proposed additional effector to establish polarised hyphal growth (Figure 8). At 37 °C, GasC also activates two pathways regulating conidial germination. In one pathway, similar to 25 °C, CflA activates PakA via the CRIB domain, and this interaction is required for PakA function. Unlike 25 °C, RasA does not activate CflA, PakA plays a crucial role during the establishment of polarised arthroconidiating hyphal growth, and no additional effector is required (Figure 8).

### The G-protein GasC Regulates Two Signaling Pathways Required for the Initiation of Filamentous Growth in P. marneffei

*gasC* encodes a heterotrimeric guanine nucleotide-binding (G-protein) α-subunit with homology to *S. cerevisiae GPA2.* Gpa2p is required for the initiation of filamentous growth (pseudohyphal growth) in response to nitrogen starvation via the activation of the cAMP-PKA pathway [2,3]. In *P. marneffei,* expression of dominant activated *gasC^G45R^* and dominant interfering *gasC^G207R^* alleles at both 25 °C and 37 °C results in accelerated or delayed conidial germination, respectively, and suggests that GasC acts as a general upstream activator initiating filamentous growth. This study suggests that GasC activates two pathways regulating germination at both 25 °C and 37 °C, one of which is dependent on PakA (Figure 8). In contrast to the accelerated germination of *gasC^G45R^* strains and the slightly delayed germination of Δ*pakA*, Δ*pakA gasC^G45R^* double mutant strains display wild-type germination at 25 °C. A reduction in the germination rates suggests that PakA is acting downstream of GasC at 25 °C. However, as germination of the Δ*pakA gasC^G45R^* strains is higher than Δ*pakA,* GasC must also regulate an additional PakA-independent pathway activating germination at 25 °C. This hypothesis is supported by the germination rates observed in the Δ*pakA gasC^G207R^* strains, which are lower than both the single mutant strains, suggesting these mutations have an additive effect. Likewise, at 37 °C, partial suppression of the Δ*pakA* germination defect by expression of the *gasC^G45R^* allele indicates that GasC acts as an upstream regulator of two pathways activating conidial germination, one of which is dependent on PakA. The activation of two pathways regulating the initiation of filamentous growth in P. marneffei differs from *S. cerevisiae.* In S. cerevisiae, the Gpa2p regulation of pseudohyphal growth occurs via the activation of the cAMP-PKA pathway, which is independent of the Ste20p/MAPK cascade [3]. The pseudohyphal growth defect of a *gpa2* mutant can be suppressed by addition of cAMP or overexpression of the dominant activated *RAS2^G19V^* allele. However, the pseudohyphal defect cannot be suppressed by the overexpression of the dominant active *STE11–4* allele and has no effect on expression of a reporter gene (*FRE-lacZ*), which is known to be regulated by the MAP kinase cascade [3]. These results together suggest that, unlike P. marneffei GasC*, S. cerevisiae*, Gpa2p regulates the cAMP pathway but not the Ste20p-MAPK cascade, thus regulating the initiation of filamentous growth.

### The Cdc42 Orthologue in P. marneffei Activates PakA during the Initiation of Filamentous Growth

In S. cerevisiae Cdc42p localises and activates Ste20p (reviewed in [12]). Therefore P. marneffei PakA is a potential downstream effector of the CflA (Cdc42p orthologue) and may be involved in similar processes. Like *cflA^D120A^* strains, which show delayed germination at 37 °C, the Δ*pakA* mutant exhibits a dramatic reduction in conidial germination at 37 °C, suggesting that PakA is crucial during the establishment of polarised growth to give rise to arthroconidiating hyphae. However, unlike CflA, PakA plays only a minor role during germination at 25 °C, as strains expressing the dominant negative *cflA^D120A^* allele show a severe delay in germination compared with only a slight delay in conidial germination for the Δ*pakA* and Δ*pakA pakA^H108G^* strains. These results suggest that PakA acts downstream of CflA at both 25 °C and 37 °C in P. marneffei. This hypothesis is also supported by the observation that the accelerated germination seen in *cflA^G14V^* strains is abrogated by the Δ*pakA* at both 25 °C and 37 °C in Δ*pakA cflA^G14V^* strains.

PAKs contain a conserved N-terminal CRIB domain and a C-terminal kinase domain (reviewed in [27]). The CRIB domain of *S. cerevisiae* Ste20p negatively inhibits the kinase domain preventing signaling [25]. This autoinhibition is relieved by interaction of the CRIB domain with Cdc42p, and this interaction is also necessary for localisation of Ste20p to sites of polarised growth [25]. The H345G mutation in the Ste20p CRIB domain shows reduced interaction with Cdc42p in a two-hybrid assay and the loss of correct localisation to sites of polarised growth. The phenotype of the *STE20^H345G^* strain is almost equivalent to the null, indicating that the interaction of Cdc42p and Ste20p is essential for Ste20p function (and for relief of autoinhibition of the kinase domain) [25]. The H108G CRIB domain mutation in P. marneffei is equivalent to the H345G of *S. cerevisiae* and was found to have a similar effect. The H108G mutation resulted in reduced localisation of PakA to sites of polarised growth and a phenotype equivalent to the deletion mutant. Compared with the accelerated germination of *cflA^G14V^* single mutant strains at both 25 °C and 37 °C, the Δ*pakA pakA^H108G^ cflA^G14V^* mutant strains exhibited reduced germination, suggesting that the interaction of CflA and PakA via the CRIB domain is required during conidial germination at both 25 °C and 37 °C. The interaction of CflA and PakA is also required during polarised growth of yeast cells, as the Δ*pakA pakA^H108G^* strains showed a swollen, abnormal yeast morphology at 37 °C, similar to the deletion strain. In S. cerevisiae, the CRIB domain is essential for pseudohyphal growth but dispensable for G protein–mediated pheromone signaling [11]. In P. marneffei the CRIB domain of PakA is also required for the initiation of filamentous growth, but unlike S. cerevisiae the initiation of filamentous growth in P. marneffei by PakA is partially dependent on G-protein signaling.

The minor role played by PakA during germination and hyphal growth at 25 °C suggests that another CflA effector, possibly a Cla4p orthologue, is required for these processes*.* The genomes of A. nidulans, M. grisea, U. maydis, Coprinopsis cinerea, Neurospora crassa, and C. albicans encode two PAKs, one with homology to Ste20p and the other to Cla4p (http://www.broad.mit.edu/annotation/fgi/). In addition to the CRIB and kinase domains of Ste20p orthologues, Cla4p homologues have a pleckstrin homology domain. Cla4p in S. cerevisiae is required for septation but does not play a role in pseudohyphal growth. However, deletion of *CLA4* in the yeasts Yarrowia lipolytica and C. albicans blocks filament formation, and the *cla4* deletion mutant of the plant pathogen U. maydis is unable to form filaments during infection [29–32].

### PAKs in P. marneffei May Have Overlapping Roles during Polarised Growth of Hyphae at 25 °C

*cflA* has been previously shown to play a pivotal role during hyphal morphogenesis with mutations resulting in grossly aberrant hyphae [24]. It was therefore expected that the Δ*pakA* strain may have a similar phenotype, albeit less severe, as CflA is proposed to interact and activate numerous effector proteins. The lack of a Δ*pakA* hyphal phenotype suggests that PakA is not required for hyphal growth. Like *P. marneffei,* deletion of the Ste20p homologues in C. albicans and U. maydis does not result in defects in hyphal morphology [33,34]. However, the co-localisation of the GFP::PakA fusion protein with actin and the localisation to the same cellular locations as CflA at nascent septation sites and to the hyphal apex suggests a role during polarised growth of hyphae [23]. In S. cerevisiae, Ste20p directly phosphorylates Bni1p, a component of the polarisome [35]. The polarisome is a protein complex, which contains Bni1p, Spa2p, Pea2p, and Bud6p, that promotes polarised morphogenesis during filamentous growth [35,36]. The Bni1p homologue, SepA, plays a conserved role in polarised growth in A. nidulans [37]. However, *A. nidulans* lacks a Pea2p homologue and the Spa2 homologue, SpaA, is only partially conserved in sequence and function, indicating that the polarisome in filamentous fungi likely consists of a modified set of components with different contributions to polarisome function [36]. The implication is that in P. marneffei, in addition to specific developmental roles, *pakA* and *pakB* play complementary, and possibly overlapping, roles in the establishment of polarised growth during conidial germination and in the maintenance of an axis of polarisation during hyphal growth. How the two PAKs coordinately regulate different aspects of development of multi-cellular fungi still remains unclear. The analysis of a *CLA4* orthologue from P. marneffei may resolve many of these issues.

## Materials and Methods

### Molecular techniques.

P. marneffei genomic DNA and RNA was isolated as previously described [38,39]. Southern and northern blotting was performed with Amersham Hybond N+ membrane according to the manufacturer's instructions. Filters were hybridized using [α−^32^P]dATP-labeled probes by standard methods [40].

### Cloning and plasmid construction.

Primers L18 (5′-TGATCCCACAAAACTTTACT-3′) and L19 (5′-GCTCGTTTCTCAGGGTCCAC-3′) were used to amplify the A. nidulans genomic sequence encoding the conserved kinase domain of the *STE20* homologue. The PCR product was sequenced and used to screen a P. marneffei genomic library (constructed in λGEM-11) at low stringency (50% formamide, 2 x SSC, 37 °C). A 6.4 kb *NotI/BglII* hybridizing fragment from a positively hybridizing clone was subcloned into *NotI/BamHI* digested pBluescript II SK^+^ (pKB5751). Sequencing was performed by the Australian Genome Research Facility and analyzed using Sequencher 3.1.1 (Gene Codes Corporation). The Genbank accession number of the *P. marneffei pakA* gene is AY621630.

A *pakA* deletion construct (pKB5792) was generated by replacing the 2.5 kb *EcoRV/ClaI* fragment of pKB5751 with the 2.5 kb *SmaI/ClaI* fragment containing the *pyrG^+^* selectable marker. This resulted in *pyrG^+^* flanked by 2.6 kb of 5′ and 1.1 kb of 3′ *pakA* sequence, and deleted from −425 to +2030*.* Inverse PCR using the mutagenic primers N30 (5′-ACATGAGTAACACCGACAGGG-3′) and N32 (5′-TGGATACGACAATCAGACTGG-3′) was used to introduce the H108G mutation into *pakA* generating pKB5908. The integrity of the construct was confirmed by sequencing.

The *gfp::pakA* and *gfp::pakA^H108G^* constructs were generated by ligating a *BamHI/XbaI* fragment from pKB5751 (*pakA*) and pKB5908 (*pakA^H108G^*) into pALX196 (*gpdA(p)::gfp*).

### Fungal strains and media.

Strains used in this study are shown in Table 1. The Δ*pakA* strain (Δ*pakA::pyrG^+^*) was generated by transforming the strain SPM4 with linearised pKB5792 and selecting for pyrG^+^. Transformation was performed using the previously described protoplast method [38]. The Δ*pakA pyrG^−^* strain was isolated by plating the Δ*pakA* strain (Δ*pakA::pyrG^+^*) on medium containing 1 mg/mL^−1^ 5-FOA supplemented with 10 mM γ-amino butyric acid (GABA) and 5 mM uracil to select for the loss of the *pyrG* marker. A 5-FOA resistant sector was isolated that had a restriction pattern consistent with loss of *pyrG* at the *pakA* locus. The strain is unable to grow in the absence of 5 mM uracil.

**Table 1:**
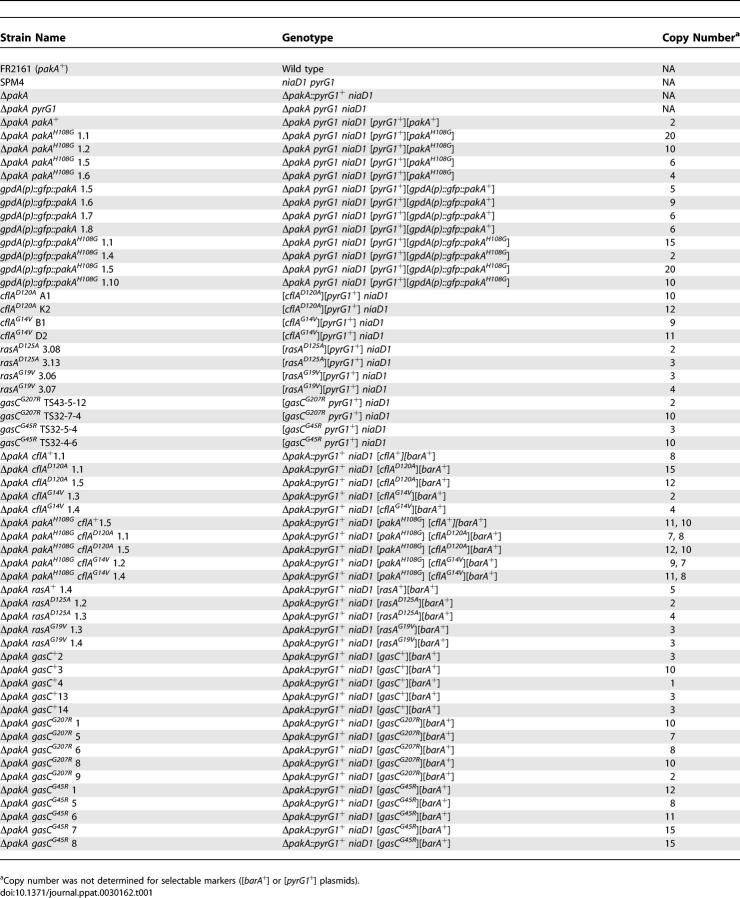
P. marneffei Strains Used in This Study

P. marneffei FRR2161, SPM4, *cflA^D120A^, cflA^G14V^, rasA^D125A^, rasA^G19V^, gasC^G207R^*, and *gasC^G45R^* have been previously described [22–24,38]. All other strains listed in Table 1 were generated by cotransformation of the Δ*pakA pyrG* or Δ*pakA* strain with plasmids containing the appropriate mutant allele and either pAB4342 (*pyrG^+^*) or pMT1612 (*barA^+^*) as selectable markers. Southern blot analysis was used to confirm cotransformation and to determine the plasmid copy number.

At 25 °C strains were grown on A. nidulans minimal medium (ANM) supplemented with 1% glucose and 10 mM GABA or on synthetic dextrose (SD) medium supplemented with 10 mM ammonium sulphate ([NH_4_]_2_SO_4_) as a sole nitrogen source [41,42]. At 37 °C, strains were grown on BHI medium or on SD medium supplemented with 10 mM (NH_4_)_2_SO_4_.

### In vivo macrophage assay.

J774 murine macrophages (1 × 10^5^) were seeded into each well of a 6-well microtitre tray containing one sterile coverslip and 2 mL of complete Dulbecco's Modified Eagle Medium (complete DMEM: DMEM, 10% fetal bovine serum, 2 mM L-glutamine and penicillin-streptomycin). Macrophages were incubated at 37 °C for 24 h before activation with 0.1μg/mL^−1^ lipopolysaccharide (LPS) from E. coli (Sigma). Macrophages were incubated a further 24 h at 37 °C and washed 3 times in phosphate buffered saline, and 2 mL of complete DMEM medium containing 1 × 10^6^ conidia was added. A control lacking conidia was also performed. Macrophages were incubated for 2 h at 37 °C (to allow conidia to be engulfed), washed once in phosphate buffered saline (to remove free conidia), and incubated a further day at 37 °C. Macrophages were fixed in 4% paraformaldehyde and stained with 1 mg/mL^−1^ fluorescent brightener 28 (calcofluor, CAL) to observe fungal cell walls. The numbers of germinated conidia was measured microscopically by counting the numbers of germinated conidia (conidia with a visible germ tube or yeast cells) in a population of approximately 100. Three independent experiments were performed. Mean and standard error of the mean values were calculated using GraphPad Prism3.

### Microscopy.

P. marneffei strains were grown on slides covered with a thin layer of solid medium, with one end resting in liquid medium [38]. Wild-type (*pakA^+^*), Δ*pakA*, Δ*pakA pakA^+^*, and Δ*pakA pakA^H108G^* strains were grown on ANM medium supplemented with GABA at 25 °C for 2 or 4 d. At 37 °C, strains were grown on BHI medium for 4 d or in liquid BHI medium for 6 d.

Immunofluorescence microscopy for examination of the actin cytoskeleton was performed using standard protocols [43]. Double staining was performed with the mouse C4 monoclonal anti-actin (Chemicon International) and rabbit anti-GFP polyclonal primary antibodies, as well as ALEXA 488 rabbit anti-mouse (Molecular Probes) and ALEXA 594 anti-rabbit (Molecular Probes) secondary antibodies. Single immunostaining controls and a minus primary antibody control were also performed. The *gfp::pakA* and *gfp::pakA^H108G^* strains were grown on agar-coated slides containing ANM plus GABA for 2 d or SD with 5 mM ammonium tartrate (NH_4_T) for 4 d at 37 °C.

Slides were examined using differential interference contrast (DIC) and epifluorescence optics for GFP, antibody fluorescence, cell wall staining with fluorescent brightener 28 (calcofluor, CAL), or nuclear staining with DAPI and viewed on a Reichart Jung Polyvar II microscope. Images were captured using a SPOT CCD camera (Diagnostic Instruments) and processed in Adobe Photoshop.

### Germination experiments.

Approximately 10^6^ spores were inoculated into 300 μL of SD plus 10 mM (NH_4_)_2_SO_4_ and incubated for 8, 12, or 15 h at 25 °C or for 8, 12, 15, or 20 h at 37 °C. The rates of germination were measured microscopically by counting the numbers of germinating conidia (conidia with a visible germ tube) in a population of 100. Three independent experiments were performed. Mean and standard error of the mean values were calculated using GraphPad Prism3. Two-level nested ANOVA was performed on the data for each time point at both 25 °C and 37 °C to test if germination rates differed significantly between genotypes and between transformants of the same genotype. ANOVA simultaneously tests two null hypotheses; there is no difference between the means of the data sets from all genotypes and there is no difference between the means of the data sets between transformants of the same genotype. The generation of two F-statistics and probability values allow rejection or acceptance of these null hypothesis at a 99% confidence. Values with an asterisk in Tables S1 and S2 showed significant differences between transformants of the same genotype.

To investigate the differential regulation of conidial germination at 25 °C and 37 °C, wild-type and Δ*pakA* conidia were incubated in liquid medium at different temperatures ranging from 25 °C to 37 °C. The 20-h incubation was performed in a gradient thermocycler using 2 °C temperature increments from 25 °C to 37 °C. The media was then transferred to a microtitre tray and the rates of germination were measured microscopically by counting the numbers of germinating conidia (conidia with a visible germ tube) in a population of 100. Three independent experiments were performed. Mean and standard error of the mean values were calculated using GraphPad Prism3.

## Supporting Information

Table S1 Percentage of Germinated Conidia at 25 °C (41 KB DOC)

Table S2 Percentage of Germinated Conidia at 37 °C (54 KB DOC)

## Abbreviations

5-FOA: 5-fluoroorotic acid
ANM: A. nidulans minimal medium
BHI: brain heart infusion
CAL: calcofluor
cAMP: cyclic adenosine monophosphate
CRIB: Cdc42/Rac interactive binding
DAPI: 4′6-diamidino-2-phenylindole
DIC: differential interference contrast
DMEM: Dulbecco's Modified Eagle Medium
GEF: guanine nucleotide exchange factor
LPS: lipopolysaccharide
MAPK: mitogen-activated protein kinase
PAK: p21-activated kinase
PKA: protein kinase A
SD: synthetic dextrose
