# Supplementary material for: A p21-Activated Kinase Is Required for Conidial Germination in Penicillium marneffei
Source: PLoS Pathog. 2007 Nov 2;3(11):e162. doi: 10.1371/journal.ppat.0030162 (PMC2048533; doi:10.1371/journal.ppat.0030162)
Supplement: Supplementary file 1 [file ppat.0030162.st001.doc]

# Supplementary Table 1. Percentage of germinated conidia at 25°C.

| **Strain** | **0 hours** | **8 hours** | **12 hours** | **15 hours** |
| --- | --- | --- | --- | --- |
| 2161 | 0.00 ± 0.00 | 2.32 ± 1.45 | 72.1 ± 3.75 | 96.2 ± 1.79 |
| Δ*pakA* | 0.00 ± 0.00 | 0.17 ± 0.17 | 33.3 ± 7.82 | 68.8 ± 3.46 |
| Δ*pakA pakA^+^* | 0.00 ± 0.00 | 0.00 ± 0.00 | 68.47 ± 4.84 | 95.2 ± 2.47 |
| Δ*pakA pakA^H108G^* 1.6 | 0.00 ± 0.00 | 0.00 ± 0.00 | 14.6 ± 4.72 | 85.9 ± 2.23 |
| Δ*pakA pakA^H108G^* 1.7 | 0.00 ± 0.00 | 0.00 ± 0.00 | 36.2 ± 2.19 | 83.7 ± 0.62 |
| *cflA^D120A^* A1 | 0.00 ± 0.00 | 0.00 ± 0.00 | 13.7 ± 16.7 | 78.8 ± 2.30 |
| *cflA^D120A^* K2 | 0.00 ± 0.00 | 0.00 ± 0.00 | 25.0 ± 8.14 | 83.5 ± 20.4 |
| *cflA^G14V^* B1 | 0.00 ± 0.00 | 1.07 ± 1.85***** | 76.7 ± 0.87 | 98.0 ± 2.67 |
| *cflA^G14V^* D2 | 0.00 ± 0.00 | 10.3 ± 4.01***** | 90.5 ± 8.96 | 94.7 ± 5.86 |
| *rasA^D125A^* 3.08 | 0.00 ± 0.00 | 0.00 ± 0.00 | 14.5 ± 0.71 | 68.3 ± 8.13 |
| *rasA^D125A^* 3.13 | 0.00 ± 0.00 | 0.00 ± 0.00 | 25.3 ±0.14 | 82.7 ± 5.16 |
| *rasA^G19V^* 3.06 | 0.00 ± 0.00 | 0.00 ± 0.00 | 12.8 ± 5.89 | 95.7 ± 1.91 |
| *rasA^G19V^* 3.07 | 0.00 ± 0.00 | 0.00 ± 0.00 | 59.6 ± 2.69 | 94.5 ± 0.57 |
| *gasC^G207R^* TS43-5-12 | 0.00 ± 0.00 | 0.00 ± 0.00 | 43.0 ± 14.1 | 88.1 ± 4.10 |
| *gasC^G207R^* TS32-7-4 | 0.00 ± 0.00 | 0.00 ± 0.00 | 43.0 ± 14.7 | 92.4 ± 3.87 |
| *gasC^G45R^* TS32-5-4 | 0.00 ± 0.00 | 0.00 ± 0.00 | 90.1 ± 1.53 | 99.2 ± 0.46 |
| *gasC^G45R^* TS32-4-6 | 0.00 ± 0.00 | 0.00 ± 0.00 | 86.8 ± 1.81 | 96.9 ± 0.85 |
| Δ*pakA cflA^+^* 1.1 | 0.00 ± 0.00 | 1.94 ± 3.37 | 52.2 ± 15.9 | 73.6 ± 14.8 |
| Δ*pakA cflA^D120A^* 1.1 | 0.00 ± 0.00 | 0.97 ±1.68 | 33.4 ±6.43 | 58.3 ± 3.86 |
| Δ*pakA cflA^D120A^* 1.5 | 0.00 ± 0.00 | 1.10 ± 1.90 | 29.9 ± 9.41 | 57.8 ±8.96 |
| Δ*pakA cflA^G14V^* 1.3 | 0.00 ± 0.00 | 2.70 ± 4.68 | 41.5 ± 2.10 | 69.4 ± 2.82 |
| Δ*pakA cflA^G14V^* 1.4 | 0.00 ± 0.00 | 1.13 ± 1.96 | 46.4 ± 1.38 | 71.6 ± 8.92 |
| Δ*pakA pakA^H108G^ cflA^+^*1.5 | 0.00 ± 0.00 | 1.27 ± 2.19 | 50.6 ± 21.0 | 66.9 ± 4.80 |
| Δ*pakA pakA^H108G^cflA^D120A^* 1.1 | 0.00 ± 0.00 | 0.52 ± 0.91 | 36.2 ± 15.5 | 54.6 ± 1.14***** |
| Δ*pakA pakA^H108G^cflA^D120A^* 1.2 | 0.00 ± 0.00 | 0.28 ± 0.48 | 18.6 ± 12.25 | 39.6 ± 1.27***** |
| Δ*pakA pakA^H108G^cflA^G14V^* 1.2 | 0.00 ± 0.00 | 12.0 ± 4.35***** | 66.0 ± 6.41 | 72.7 ± 8.78***** |
| Δ*pakA pakA^H108G^cflA^G14V^* 1.3 | 0.00 ± 0.00 | 1.14 ± 0.42***** | 38.3 ± 6.76 | 49.7 ± 3.21***** |
| Δ*pakA rasA^+^* 1.4 | 0.00 ± 0.00 | 2.18 ± 3.78 | 62.4 ± 10.1 | 67.9 ± 0.83 |
| Δ*pakA rasA^D125A^* 1.2 | 0.00 ± 0.00 | 1.26 ± 1.56 | 32.5 ± 7.82 | 59.8 ± 4.48 |
| Δ*pakA rasA^D125A^* 1.3 | 0.00 ± 0.00 | 1.58 ± 1.64 | 35.5 ± 9.00 | 73.4 ± 5.20 |
| Δ*pakA rasA^G19V^* 1.3 | 0.00 ± 0.00 | 0.83 ± 0.76 | 17.8 ± 8.27 | 42.5 ± 10.5***** |
| Δ*pakA rasA^G19V^* 1.4 | 0.00 ± 0.00 | 0.74 ± 0.75 | 34.2 ± 4.14 | 63.6 ± 8.38***** |
| Δ*pakA gasC^+^* 2 | 0.00 ± 0.00 | 0.00 ± 0.00 | 27.0 ± 12.7 | 78.4 ± 8.12 |
| Δ*pakA gasC^+^* 3 | 0.00 ± 0.00 | 0.00 ± 0.00 | 29.6 ± 11.8 | 80.9 ± 5.53 |
| Δ*pakA gasC^+^* 4 | 0.00 ± 0.00 | 0.00 ± 0.00 | 25.1 ± 11.1 | 73.4 ± 2.72 |
| Δ*pakA gasC^+^* 13 | 0.00 ± 0.00 | 0.00 ± 0.00 | 21.9 ± 14.4 | 77.6 ± 2.96 |
| Δ*pakA gasC^+^* 14 | 0.00 ± 0.00 | 0.00 ± 0.00 | 28.0 ± 6.13 | 72.2 ± 14.1 |
| Δ*pakA gasC^G207R^* 1 | 0.00 ± 0.00 | 0.00 ± 0.00 | 17.67 ± 1.60 | 38.6 ± 8.38 |
| Δ*pakA gasC^G207R^* 5 | 0.00 ± 0.00 | 0.00 ± 0.00 | 35.8 ± 11.5 | 74.8 ± 19.3 |
| Δ*pakA gasC^G207R^* 6 | 0.00 ± 0.00 | 0.00 ± 0.00 | 12.7 ± 2.67 | 45.8 ± 7.75 |
| Δ*pakA gasC^G207R^* 8 | 0.00 ± 0.00 | 0.00 ± 0.00 | 11.3 ± 4.12 | 47.1 ± 10.9 |
| Δ*pakA gasC^G207R^* 9 | 0.00 ± 0.00 | 0.00 ± 0.00 | 31.9 ± 7.92 | 57.4 ± 0.61 |
| Δ*pakA gasC^G45R^* 1 | 0.00 ± 0.00 | 0.00 ± 0.00 | 63.1 ± 20.3 | 98.8 ± 1.42 |
| Δ*pakA gasC^G45R^* 5 | 0.00 ± 0.00 | 0.00 ± 0.00 | 58.7 ± 20.3 | 96.4 ± 3.27 |
| Δ*pakA gasC^G45R^* 6 | 0.00 ± 0.00 | 0.00 ± 0.00 | 61.2 ± 18.1 | 93.9 ± 6.60 |
| Δ*pakA gasC^G45R^* 7 | 0.00 ± 0.00 | 0.00 ± 0.00 | 61.1 ± 19.3 | 91.5 ± 1.10 |
| Δ*pakA gasC^G45R^* 8 | 0.00 ± 0.00 | 0.00 ± 0.00 | 57.9 ± 13.4 | 96.1 ± 5.12 |

Values shown are the mean ± standard error of the mean for 3 independent experiments.

Significant differences between germination rates of transformants of the same genotype, as determined by ANOVA analysis, are marked (*).
