# Supplementary material for: A p21-Activated Kinase Is Required for Conidial Germination in Penicillium marneffei
Source: PLoS Pathog. 2007 Nov 2;3(11):e162. doi: 10.1371/journal.ppat.0030162 (PMC2048533; doi:10.1371/journal.ppat.0030162)
Supplement: Supplementary file 2 [file ppat.0030162.st002.doc]

# Supplementary Table 2. Percentage of germinated conidia at 37°C.

| **Strain** | **0 hours** | **8 hours** | **12 hours** | **15 hours** | **20 hours** |
| --- | --- | --- | --- | --- | --- |
| 2161 | 0.00 ± 0.00 | 0.64 ± 0.64 | 29.8 ± 3.77 | 46.4 ± 3.14 | 66.4 ± 2.53 |
| Δ*pakA* | 0.00 ± 0.00 | 0.00 ± 0.00 | 0.00 ± 0.00 | 1.24 ± 0.41 | 1.40 ± 0.48 |
| Δ*pakA pakA^+^* | 0.00 ± 0.00 | 0.00 ± 0.00 | 24.9 ± 2.43 | 43.9 ± 2.66 | 63.3 ± 7.10 |
| Δ*pakA pakA^H108G^* 1.6 | 0.00 ± 0.00 | 0.00 ± 0.00 | 0.33 ± 0.33 | 7.71 ± 1.81 | 8.34 ± 0.55 |
| Δ*pakA pakA^H108G^* 1.7 | 0.00 ± 0.00 | 0.27 ± 0.27 | 0.00 ± 0.00 | 2.98 ± 0.82 | 4.60 ± 1.69 |
| *cflA^D120A^* G66 | 0.00 ± 0.00 | 0.00 ± 0.00 | 2.57 ± 3.69***** | 49.3 ± 18.9 | 61.8 ± 14.4 |
| *cflA^D120A^* G68 | 0.00 ± 0.00 | 0.00 ± 0.00 | 18.0 ± 10.1***** | 52.3 ± 14.4 | 56.2 ± 16.8 |
| *cflA^G14V^* G71 | 0.00 ± 0.00 | 1.65 ± 1.75 | 55.2 ± 1.21***** | 81.9 ± 1.16 | 88.8 ± 5.44 |
| *cflA^G14V^* G72 | 0.00 ± 0.00 | 2.78 ± 2.42 | 74.6 ± 11.8***** | 90.4 ± 5.03 | 91.4 ± 7.26 |
| *rasA^D125A^* 3.08 | 0.00 ± 0.00 | 0.00 ± 0.00 | 34.8 ± 5.02 | 60.6 ± 5.16 | 52.2 ± 27.8 |
| *rasA^D125A^* 3.13 | 0.00 ± 0.00 | 0.00 ± 0.00 | 48.8 ± 11.7 | 54.0 ± 7.57 | 52.0 ± 8.20 |
| *rasA^G19V^* 3.06 | 0.00 ± 0.00 | 0.00 ± 0.00 | 30.2 ± 3.75 | 33.7 ± 7.07 | 67.0 ± 15.8 |
| *rasA^G19V^* 3.07 | 0.00 ± 0.00 | 0.00 ± 0.00 | 43.2 ± 0.35 | 40.5 ± 8.77 | 61.2 ± 2.40 |
| *gasC^G207R^* TS43-5-12 | 0.00 ± 0.00 | 0.00 ± 0.00 | 5.53 ± 1.35 | 20.6 ± 2.48 | 30.5 ± 2.51 |
| *gasC^G207R^* TS32-7-4 | 0.00 ± 0.00 | 0.00 ± 0.00 | 6.78 ± 2.12 | 34.8 ± 18.2 | 60.6 ± 18.7 |
| *gasC^G45R^* TS32-5-4 | 0.00 ± 0.00 | 0.00 ± 0.00 | 87.8 ± 2.27 | 96.6 ± 1.06 | 96.1 + 1.95 |
| *gasC^G45R^* TS32-4-6 | 0.00 ± 0.00 | 0.00 ± 0.00 | 88.1 ± 2.47 | 91.8 ± 2.91 | 96.5 ± 1.84 |
| Δ*pakA cflA^+^* 1.1 | 0.00 ± 0.00 | 0.00 ± 0.00 | 0.00 ± 0.00 | 1.95 ± 1.58 | 2.00 ± 0.47 |
| Δ*pakA cflA^D120A^* 1.1 | 0.00 ± 0.00 | 0.00 ± 0.00 | 0.00 ± 0.00 | 1.05 ± 0.70 | 0.66 ± 0.12 |
| Δ*pakA cflA^D120A^* 1.5 | 0.00 ± 0.00 | 0.00 ± 0.00 | 0.00 ± 0.00 | 1.39 ± 1.77 | 1.54 ± 0.12 |
| Δ*pakA cflA^G14V^* 1.3 | 0.00 ± 0.00 | 0.00 ± 0.00 | 0.00 ± 0.00 | 0.54 ± 0.48 | 1.58 ± 0.37 |
| Δ*pakA cflA^G14V^* 1.4 | 0.00 ± 0.00 | 0.00 ± 0.00 | 0.00 ± 0.00 | 0.67 ± 0.62 | 1.80 ± 1.49 |
| Δ*pakA pakA^H108G^ cflA^+^*1.5 | 0.00 ± 0.00 | 0.00 ± 0.00 | 0.00 ± 0.00 | 0.79 ± 0.13 | 0.45 ± 0.39 |
| Δ*pakA pakA^H108G^ cflA^D120A^* 1.1 | 0.00 ± 0.00 | 0.00 ± 0.00 | 0.00 ± 0.00 | 1.56 ± 0.75 | 3.13 ± 0.65 |
| Δ*pakA pakA^H108G^ cflA^D120A^* 1.2 | 0.00 ± 0.00 | 0.00 ± 0.00 | 0.00 ± 0.00 | 0.48 ± 0.43 | 0.86 ± 0.22 |
| Δ*pakA pakA^H108G^ cflA^G14V^* 1.2 | 0.00 ± 0.00 | 0.33 ± 0.58 | 2.80 ± 2.82 | 4.33 ± 1.14 | 3.99 ± 3.20 |
| Δ*pakA pakA^H108G^ cflA^G14V^* 1.3 | 0.00 ± 0.00 | 0.00 ± 0.00 | 1.39 ± 0.87 | 0.58 ± 0.52 | 0.45 ± 0.78 |
| Δ*pakA rasA^+^* 1.4 | 0.00 ± 0.00 | 0.00 ± 0.00 | 0.00 ± 0.00 | 0.49 ± 0.42 | 0.94 ± 0.82 |
| Δ*pakA rasA^D125A^* 1.2 | 0.00 ± 0.00 | 0.00 ± 0.00 | 0.00 ± 0.00 | 0.93 ± 0.90 | 1.54 ± 0.50 |
| Δ*pakA rasA^D125A^* 1.3 | 0.00 ± 0.00 | 0.00 ± 0.00 | 0.00 ± 0.00 | 1.70 ± 1.55 | 2.02 ± 0.52 |
| Δ*pakA rasA^G19V^* 1.3 | 0.00 ± 0.00 | 0.00 ± 0.00 | 0.00 ± 0.00 | 1.06 ± 1.01 | 0.89 ± 0.5 |
| Δ*pakA rasA^G19V^* 1.4 | 0.00 ± 0.00 | 0.00 ± 0.00 | 0.00 ± 0.00 | 0.47 ± 0.46 | 2.04 ± 0.53 |
| Δ*pakA gasC^+^* 2 | 0.00 ± 0.00 | 0.00 ± 0.00 | 0.00 ± 0.00 | 0.69 ± 0.12 | 1.15 ± 0.39 |
| Δ*pakA gasC^+^* 3 | 0.00 ± 0.00 | 0.00 ± 0.00 | 0.00 ± 0.00 | 2.36 ± 2.48 | 5.05 ± 3.89 |
| Δ*pakA gasC^+^* 4 | 0.00 ± 0.00 | 0.00 ± 0.00 | 0.00 ± 0.00 | 0.64 ± 0.56 | 1.19 ± 0.49 |
| Δ*pakA gasC^+^* 13 | 0.00 ± 0.00 | 0.00 ± 0.00 | 0.00 ± 0.00 | 1.54 ± 1.29 | 2.31 ± 1.34 |
| Δ*pakA gasC^+^* 14 | 0.00 ± 0.00 | 0.00 ± 0.00 | 0.00 ± 0.00 | 1.11 ± 0.65 | 3.01 ± 1.38 |
| Δ*pakA gasC^G207R^* 1 | 0.00 ± 0.00 | 0.00 ± 0.00 | 0.00 ± 0.00 | 0.00 ± 0.00 | 0.60 ± 0.52 |
| Δ*pakA gasC^G207R^* 5 | 0.00 ± 0.00 | 0.00 ± 0.00 | 0.00 ± 0.00 | 0.70 ± 0.69 | 2.70 ± 1.68 |
| Δ*pakA gasC^G207R^* 6 | 0.00 ± 0.00 | 0.00 ± 0.00 | 0.00 ± 0.00 | 0.00 ± 0.00 | 0.51 ± 0.45 |
| Δ*pakA gasC^G207R^* 8 | 0.00 ± 0.00 | 0.00 ± 0.00 | 0.00 ± 0.00 | 0.51 ± 0.47 | 0.61 ± 0.53 |
| Δ*pakA gasC^G207R^* 9 | 0.00 ± 0.00 | 0.00 ± 0.00 | 0.00 ± 0.00 | 0.52 ± 0.46 | 0.55 ± 0.48 |
| Δ*pakA gasC^G45R^* 1 | 0.00 ± 0.00 | 0.00 ± 0.00 | 0.00 ± 0.00***** | 10.5 ± 1.47 | 21.3 ± 2.83 |
| Δ*pakA gasC^G45R^* 5 | 0.00 ± 0.00 | 0.00 ± 0.00 | 0.47 ± 0.82***** | 12.8 ± 4.97 | 21.4 ± 2.82 |
| Δ*pakA gasC^G45R^* 6 | 0.00 ± 0.00 | 0.00 ± 0.00 | 11.1 ± 6.82***** | 19.4 ± 12.5 | 29.9 ± 15.6 |
| Δ*pakA gasC^G45R^* 7 | 0.00 ± 0.00 | 0.00 ± 0.00 | 0.57 ± 0.99***** | 18.5 ± 19.8 | 26.5 ± 20.0 |
| Δ*pakA gasC^G45R^* 8 | 0.00 ± 0.00 | 0.00 ± 0.00 | 1.85 ± 1.76***** | 10.5 ± 2.35 | 12.6 ± 3.32 |

Values shown are the mean ± standard error of the mean for 3 independent experiments.

Significant differences between germination rates of transformants of the same genotype, as determined by ANOVA analysis, are marked (*).
